# Non-destructive high-throughput measurement of elastic-viscous properties of maize using a novel ultra-micro sensor array and numerical validation

**DOI:** 10.1038/s41598-023-32130-5

**Published:** 2023-03-25

**Authors:** Taiken Nakashima, Haruka Tomobe, Takumi Morigaki, Mengfan Yang, Hiroto Yamaguchi, Yoichiro Kato, Wei Guo, Vikas Sharma, Harusato Kimura, Hitoshi Morikawa

**Affiliations:** 1 Hokkaido University, Sapporo, Japan; 2 Tokyo Institute of Technology, Tokyo, Japan; 3 University of Tokyo, Tokyo, Japan; 4 Kyoto University, Kyoto, Japan

**Keywords:** Plant breeding, High-throughput screening

## Abstract

Maize is the world's most produced cereal crop, and the selection of maize cultivars with a high stem elastic modulus is an effective method to prevent cereal crop lodging. We developed an ultra-compact sensor array inspired by earthquake engineering and proposed a method for the high-throughput evaluation of the elastic modulus of maize cultivars. A natural vibration analysis based on the obtained Young’s modulus using finite element analysis (FEA) was performed and compared with the experimental results, which showed that the estimated Young’s modulus is representative of the individual Young’s modulus. FEA also showed the hotspot where the stalk was most deformed when the corn was vibrated by wind. The six tested cultivars were divided into two phenotypic groups based on the position and number of hotspots. In this study, we proposed a non-destructive high-throughput phenotyping technique for estimating the modulus of elasticity of maize stalks and successfully visualized which parts of the stalks should be improved for specific cultivars to prevent lodging.

## Introduction

The Green Revolution has increased the yield of cereals by optimizing varieties, soil nutrients, weed and pest management, and irrigation system^1–3^. However, the lodging of plants due to the wind and rain has been a critical factor in limiting the yield of cereals over the past half-century. In the Green Revolution, semi-dwarf varieties with higher harvest index were opted to avoid the lodging, instead of those with larger and stronger plants that could withstand lodging. Now we are back at this fork in the road. As harvest indices in many cereal crops have reached at theoretical maxima, our next step is to systematically produce lines with higher biomass production and enhanced lodging resistance to meet increasing food demand.

Lodging is a mechanically complex problem; if the wind force is balanced with the bearing force, the stem structure will not collapse. However, if the wind force is greater, the stem collapses^1,4^. The relationship between the wind velocity and the aerodynamic forces exerted on the crop is complex, but is believed to be roughly proportional^5,6^. The power spectrum observed in previous studies shows that most of the power is concentrated in the long-period region of wind frequency above 1 Hz, with little power in the short-period region below 1 Hz^7^. Furthermore, the stress–strain constitutive relationship between the stress and displacement fields of the crop, in general, is a nonlinear and depends on shape and material of crop. However, several studies have demonstrated that, in situations where the deformation is small and the geometry is simple, the constitutive relationship in plant stem is purely linear^1,3,8–12^. Plants are complex in shape and vary in strength from tissue to tissue, and thus exhibit complex opportunistic properties even under low deformation^13,14^.

Ookawa et al.^1^ modeled the stress–strain relationship of rice plants based on quasi-static mechanics and found that the Young’s modulus and cross-sectional secondary moment independently govern the resistance of stem to collapse. The authors spent over ten years cloning genes and conducting stem breakage tests on many recombinant inbred lines (RILs) to estimate Young’s modulus and cross-sectional secondary moment. Eventually, they developed numerous varieties with a high Young’s modulus and secondary moment of the section, achieving both high biomass production and lodging resistance in rice.

Nevertheless, applying this approach to maize is difficult for the following reasons. (i), as the height of a maize plant is 2–3 m, a considerable amount of labor is required to sample the stalks and measure Young's modulus using bending tests^8^ even for a few maize plants. (ii) Previous studies have shown that maize, as well as rice, can have hundreds of RILs^15–17^, which inevitably results in a high cost for phenotyping. (iii) Unlike rice, the Young’s modulus and cross-sectional shape of maize vary greatly among nodes in a stem; therefore, the Young’s modulus at one location may not represent the Young’s modulus of another location within the same individual^18^. Keeping the above challenges in view, an accurate and high-throughput method to measure the Young’s modulus of maize is required to breed maize lines with both high biomass productivity and high resistance to lodging.

As mentioned above, the maize body and the ground can be considered linear elastic bodies when the deformation is small^15–17,19–21^. Therefore, it seems possible to instantaneously estimate the elastic properties of the maize plant by placing a group of sensors on the surface of the maize body and measuring the microtremor simultaneously.

This technique has been successfully employed in earthquake engineering^19–26^. However, in earthquake engineering, the spacing between sensors ranges from a few meters to several kilometers. This is mainly because the objective is to measure the elastic modulus of an area with a radius of several tens of meters to several kilometers^19–21,23–25,27^. For this reason, a single sensor generally weighs from several 100 g to five kilograms and has a sampling frequency of less than 100 Hz^19–21^. Recently, Taylor and coworkers used an apparatus to measure the elastic modulus of soil samples from P- and S-wave velocities, which necessitated a 1 kHz sampling ratio to capture the phase^22^ using heavy piezometers.

Young's modulus of plants ranges from 0.1 to 100 MPa, which requires high-frequency sampling 500 Hz to 1 kHz, to estimate the elastic modulus of maize using a sensor with a mass of 2–3 g or less. In addition, the sensor should be ultra-compact and lightweight so that it does not deform the plant and influence its vibration characteristics. Needless to state, the sensors employed in earthquake engineering cannot be directly used to identify the elastic modulus of living maize plants due to their excessive weight, large size, and low sampling frequency.

In this study, we developed a method for the simultaneous multi-point measurement of maize surface waves using a group of ultra-compact sensors and a high-throughput Young’s modulus measurement method. The sensors employed in this study are based on micro-electro-mechanical systems (MEMS)^28–30^, which weigh less than a few grams and are thus unlikely to affect the vibrational characteristics of the maize body. In the proposed method, three MEMS sensors are placed on the corn stalk for spatial–temporal measurements of acceleration waveforms while applying an external impact to the plant. The time required for a single measurement is only approximately 2 s, and the small size and portability of the device enable extremely high-throughput measurements. Subsequently, this paper provides the details of the data interpretation technique for a high-throughput simultaneous estimate of Young's modulus, the plant’s natural frequency.

In addition, to verify the validity of the measured Young's modulus and natural frequencies, elastodynamics simulations of maize plants were performed using finite element analysis (FEA)^31–35^. In these simulations, based on the estimated Young’s modulus and the morphological information of corn, the natural frequencies of the above-ground part of maize are predicted. By comparing the simulation results with the field measurement, we verified that the measured Young’s modulus is consistent with the measured natural frequencies.

## Results

### Estimating hot-spots of elastic stress under wind-induced vibration in maize using FEA

**Figure 1:**
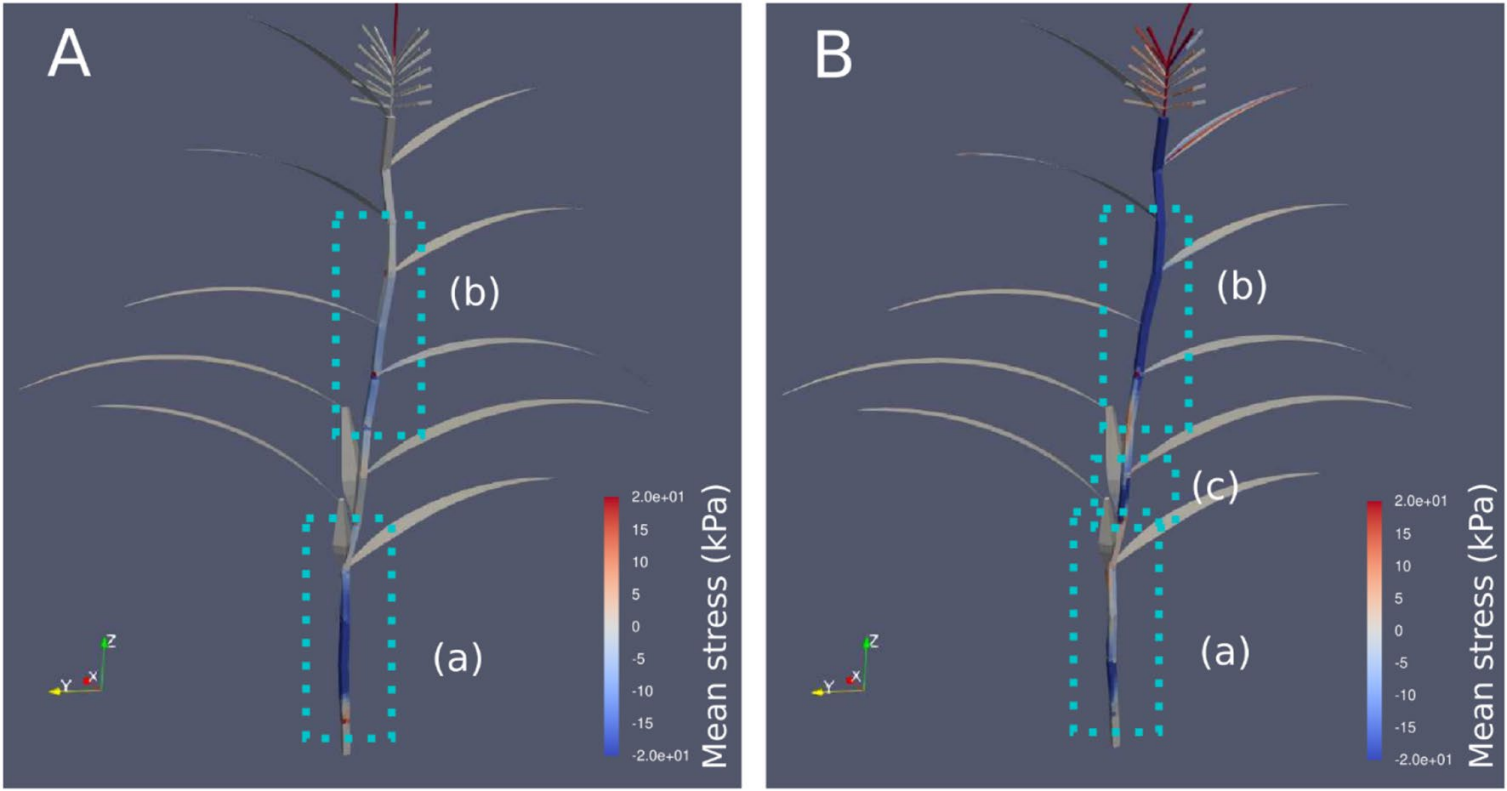
Preliminary finite element analysis (FEA) of a maize plant: (**A**) contour map of the mean stress at the first eigenmode, and (**B**) the second eigenmode. The results indicate that that the below-ear (inset a) and above-ear sections (inset b) deform significantly under free vibration. Thus, the Young’s modulus of these two regions may contribute essentially to the plant’s mechanical properties.

Preliminary FEA was performed to determine the most critical parts of the stem. In this preliminary analysis, the stem diameter and length were assumed to be 20 mm and 1.8 m, respectively, using 2021 data for the cultivar KD580 as a reference. Figure 1 shows a spatial distribution of the mean stress in the first and second eigenmode. In the first eigenmode, the stem vibrates in the direction parallel to the rows (Fig. 1A) whereas in the second eigenmode the stem vibrates perpendicular to the rows (Fig. 1B). This analysis revealed the existence of two hot-spots, which are located below (refer to inset (a) in Fig. 1) and above (refer to inset (b) in Fig. 1) the ear on the maize stem. In addition, in the case of the second eigenmode, a slight hot-spot appears in inset (c). It is well known that a stem has a single hot-spot when it behaves like an elastic cantilever beam^11^. However, the maize stem has two or three hotspots, which indicates that the maize plant body is not similar to a cantilever, and leaves and ears play essential roles under free vibration. The sensors were arranged in such a manner that they straddled parts (a) and (b).

### Measurement of Young’s modulus from the S-wave velocity

**Figure 2:**
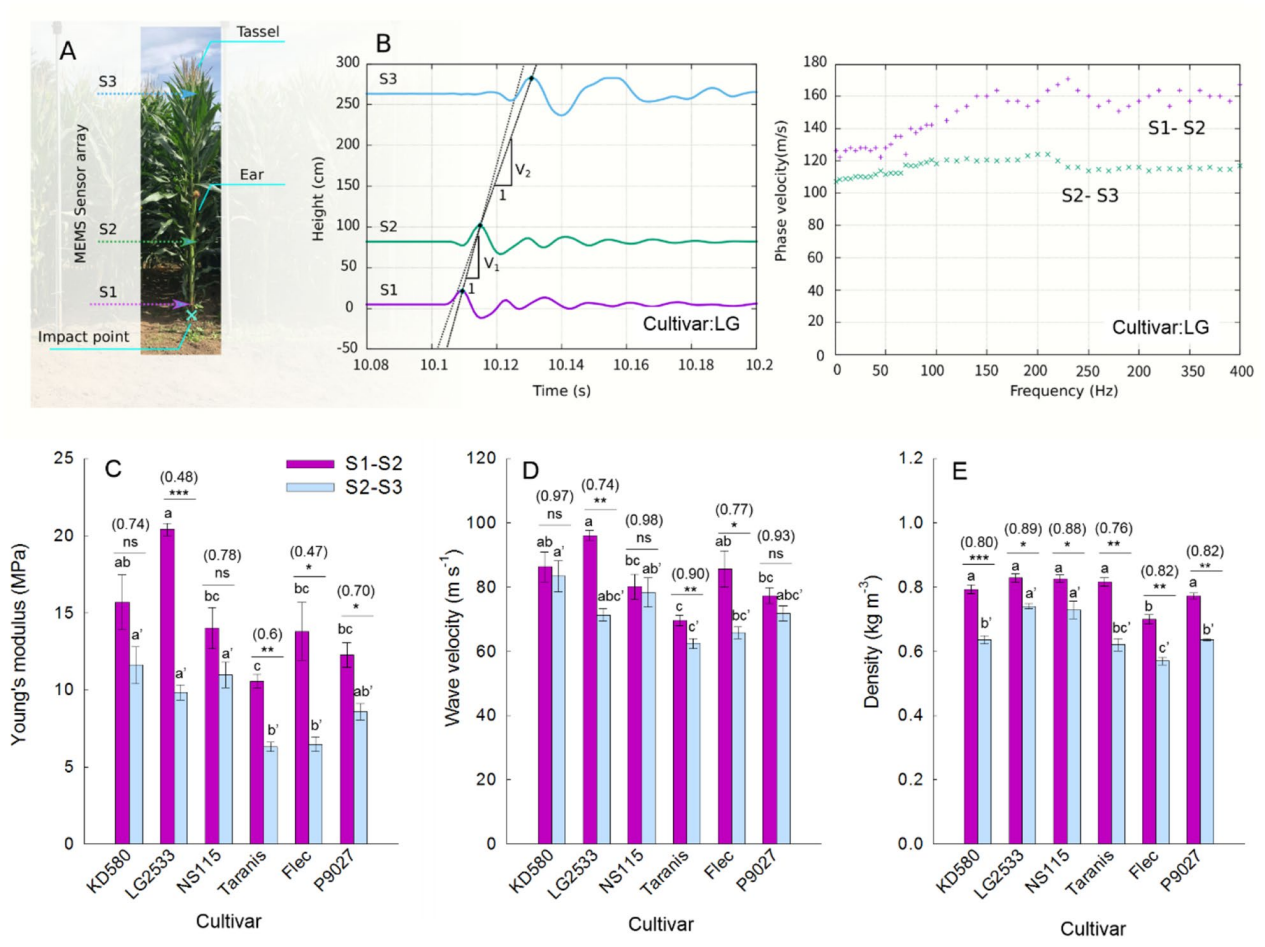
Record section and Young’s modulus of the vibration data associated with the impact test: (**A**) Position of the micro-electro-mechanical systems (MEMS) sensor array attached to the matured maize plant (cv. LG2533). An impulsive load wave was applied at the bottom of the stem to generate the surface shear waves. (**B**) Travel time curve for the shear wave (left) across the maize surface (cv. LG2533) and the dispersion curve (right). The travel time curve (solid line) clearly exhibits the different wave velocity in the stem, V1 between S1 to S2 and V2 between S2 and S3. Dispersion curve indicates the presence of a Lamb wave in section S1 to S2, while very small dispersion is present in section S2–S3. (**C**) Young’s modulus estimated by V_1_ and V_2_ based on the linear elasticity. (**D**) Wave velocities and (**E**) stalk densities below (S1–S2) and above (S2–S3) the ear for different cultivars. Values in C–E are means ± se (n = 4). Tukey’s HSD test was used to examine the statistical significances at* p* < 0.05 and different alphabets indicate significant differences. *, **, and *** in (**C**) indicate significant differences between below- and above-ear stalk sections at *p* < 0.05, *p* < 0.01, *p* < 0.001, respectively, by two-tailed paired Student’s t-test. Values in parentheses above the bars are the ratio of above to below ear sections.

Based on the FEA results, three MEMS accelerometers were arranged at locations S1, S2 and S3, as depicted in Fig. 2A. Seismic waves were excited by striking the impact point at the ground edge with a hammer, as shown in Fig. 2A. Elastic transverse waves (read as S-wave) were generated by impacting the bottom part of the stem with a hammer. Figure 2B plots the travel time curves of acceleration waves in the horizontal direction, where the horizontal axis denotes the time, and the vertical axis denotes the location of the sensors. It is evident from this figure that the elastic wave passes through the sensors S1, S2, and S3 in that order. Subsequently, the tests were conducted on six cultivars (viz., KD580, LG2533, NS115, Taranis, Flec and P9027) with various grass types, all of which showed two different elastic wave velocities in the S1-S2 and S2-S3 sections, as illustrated in Fig. 2B.

For S-wave, the Young’s modulus $E$(kPa) can be estimated from the S-wave velocity, ${V}_{s}$ (m/s) by

$$E=2\left(1+\nu \right)\rho {\left({V}_{s}\right)}^{2}$$

where $\nu$ and $\rho$ denote the Poisson’s ratio and the density of the elastic medium (t/m^3^), respectively. It is noteworthy that the above relationship is exact when the S-wave is a body wave and approximately holds when the S wave is a surface wave. The waveform raises an important question as to whether this wave can be regarded as a body wave. The results for all cultivars indicate that the wave period was approximately 8 ms, which is an average of ones on all cultivars/plants, and the S-wave velocity was approximately 80 m/s.

From period $T$ and wave speed $c$, the wavelength $\lambda$ can be obtained as follows:

$$\lambda =cT$$

Thus, the wavelengths were determined to be approximately 64 cm, which is approximately 50 times larger than the radius of the stem (1.2–1.4 cm). Waves traveling through thin plates are called Lamb waves^36^. Lamb waves propagate while reflecting off a thin elastic plate and exhibit dispersion phenomenon wherein wave speed depends on the frequency. Owing to this dispersion, the wave speed of the Lamb wave was slightly slower than that of the body wave.

Here, Young’s modulus is obtained by assuming that the observed wave velocity is approximately the same as the S-wave velocity, as shown in Fig. 2B. The wave reversion in Fig. 2B indicates the presence of the Lamb wave because the Lamb wave has a dispersion curve, as shown in previous investigations^36,37^. A typical Lamb wave has a small shear wave velocity at low frequencies, as shown in Fig. 2B. Considering that the body S-wave and Lamb wave have different wave velocities, the following subsection examines whether this estimated Young’s modulus is consistent with the overall natural frequency. Figure 2C shows the Young’s modulus estimates for the S1–S2 and S2–S3 sections for each cultivar.

Significant differences were observed between the Young’s modulus and the wave velocities of below-ear (*E*_1_) and above-ear sections (*E*_2_) (Fig. 2D,E). Measurements ranged from 10.6 to 20.4 MPa for *E*_1_ and from 6.3 to 11.6 MPa for *E*_2_. In all cultivars, *E*_1_ was higher than *E*_2_, suggesting that the stalk section below the ear was stiffer than that above the ear, although no significant difference was observed between *E*_1_ and *E*_2_ in the KD580 and NS115.

The highest *E*_1_ was observed in the high-yielding and lodging-tolerant LG2533 cultivar, while the lowest value was observed in the high-yielding but more lodging-susceptible Taranis cultivar. LG2533 also showed a lower *E*_2_/*E*_1_ ratio than the other cultivars examined, except for Flec, which is another cultivar with high lodging tolerance. Therefore, a *E*_2_/*E*_1_ ratio below 0.5 in these two cultivars is an indicator of stiffer basal and flexible upper stalk sections, which mechanically resemble a fishing rod. Such physical properties of stalks are potentially favorable traits for withstanding heavy wind loads on the upper canopy with increased stalk plasticity.

Regarding the two theoretical determinants of Young’s modulus, the observed varietal differences in *E*_2_/*E*_1_ ratio were largely attributed to the variation in wave velocities rather than to the volumetric density of the stalk (Fig. 2C,E). The lodging-tolerant cultivars LG2533 and Flec also showed the lowest *Vs*_1_/*Vs*_2_ ratios, suggesting that non-destructive measurements of *V*s on below- and upper-ear stalk provide a rough estimation of the *E*_2_/*E*_1_ ratio in a high-throughput manner without destructive sampling of stalk density.

### Measurement of eigenfrequency and damping ratio of maize stem

The natural frequencies of individual maize plants were measured to verify the correctness of the Young’s modulus obtained in the previous subsection and to investigate the possibility of inverse estimation of Young’s modulus from natural frequencies. As discussed in the previous subsection, the Young’s modulus estimated based on the wave velocity may contain errors owing to differences in the velocities of the Lamb and body S-waves. Therefore, the natural frequencies at the individual scale were estimated and numerical calculations were performed to verify the validity of the estimated Young’s modulus. It is noteworthy that Nakata et al.^9^ developed a method to estimate the Young’s modulus of stems from the natural frequencies of *Arabidopsis*. Further, it is opined that if such technique, if applicable to the maize in the field, would be more efficient than the present MEMS sensors-based procedure.

**Figure 3:**
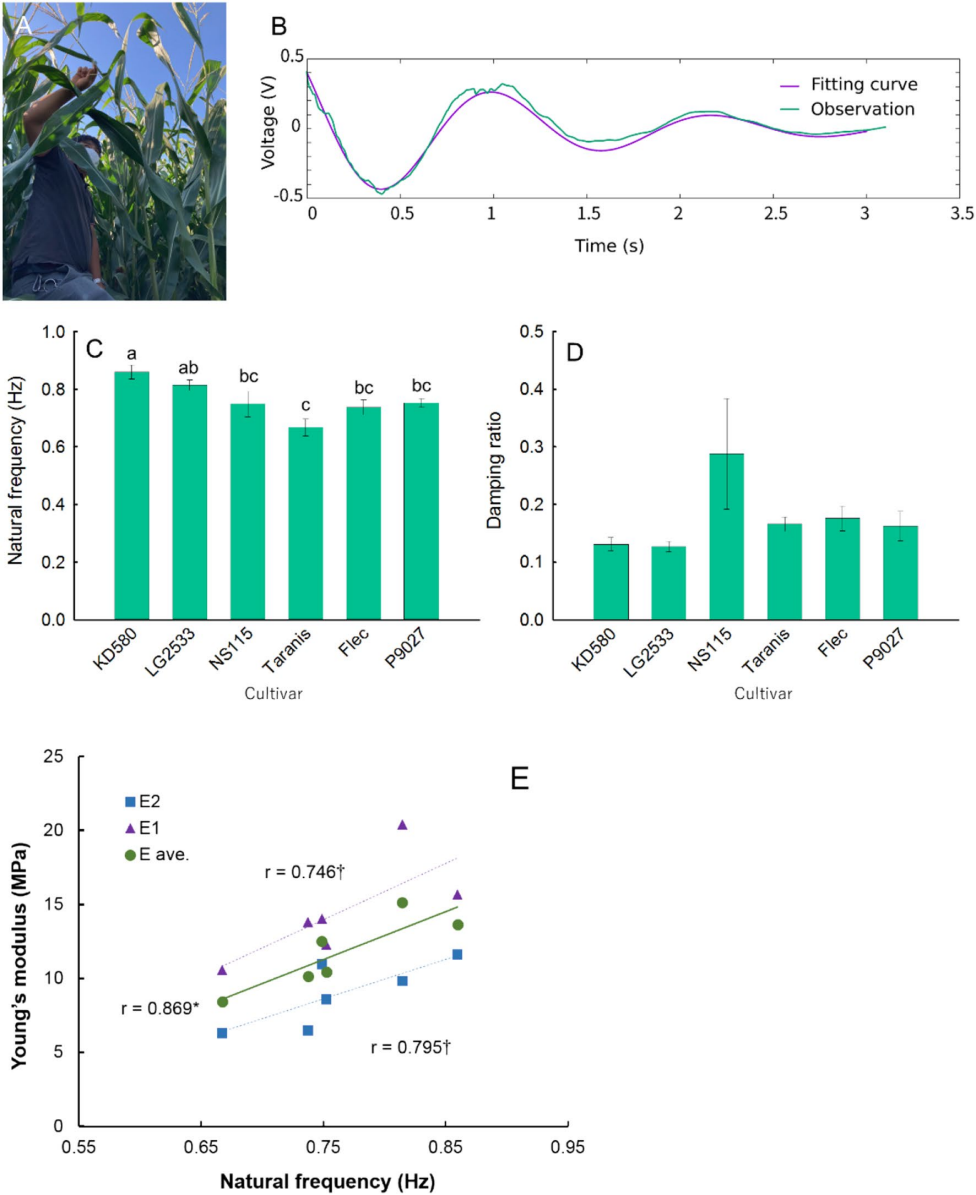
Experimentally determined natural frequency and damping ratio. (**A**) Bending vibration test for measuring the step response associated with the bending mode. (**B**) The vibration data were bandpass filtered at 0.1–10.0 Hz to remove noise. Subsequently, the response was fit by the analytical solution of the damped free vibration problem. (**C**, **D**) The natural frequency and the damping ratio obtained from the fitting of the observation. (**E**) The relationship between natural frequency and Young’s modulus. Values in C and D are means ± s.e. (standard error; n = 4). Tukey’s HSD test was used to examine the statistical significances at *p* < 0.05 and different alphabets indicate significant differences. † and * indicate statistical significance in correlation coefficient at *p* < 0.1 and *p* < 0.05, respectively.

The bending vibration tests were performed on the maize plant in the field (Fig. 3A), wherein, at first, the Tassel was pulled and opened in the direction perpendicular to the row, and then released to set the plant in motion. The response of the maize was measured by the MEMS sensor which is placed at S3 position (Fig. 3B). The response of the plant stands for the response of an elastic body to a step loading. A step response waveform can be obtained, as illustrated in Fig. 3B. Subsequently, the measured response was processed by applying a 0.1–10.0 Hz bandpass filter, and data fitting by stochastic gradient descent method. Furthermore, the natural frequency and damping ratio of the plant are obtained by fitting the step response function of the damped vibration system.

The estimated natural frequencies and damping ratios are presented in Fig. 3C. The natural frequencies estimated using the bending vibration test differed significantly among cultivars. Taranis, a lodging-sensitive cultivar, showed a significantly lower natural frequency than KD580 and LG2533 that exhibited the highest Young’s modulus of the below-ear stalk section. In contrast to the natural frequency, no significant varietal difference was observed in the damping ratio (Fig. 3D). However, KD580 and LG2533 showed slightly lower values than those of the other cultivars. Additionally, NS115 exhibited the highest damping ratio, which could be due to a mechanism involved in the high lodging tolerance of this cultivar.

The relationship between the Young’s modulus and natural frequency is shown in Fig. 3E. Although the Young's modulus in the S1–S2 interval and that in the S2–S3 interval showed a correlation with the natural frequencies, it was not possible to estimate the Young’s modulus from the natural frequencies. Therefore, it is difficult to accurately estimate the Young’s modulus of maize from its natural frequency, unlike with *Arabidopsis*. The damping ratio was successfully evaluated as a byproduct. High viscous damping allows wind energy to be dissipated more efficiently, making high-damping traits a potential breeding target.

### Numerical simulation of free vibration of maize

**Figure 4:**
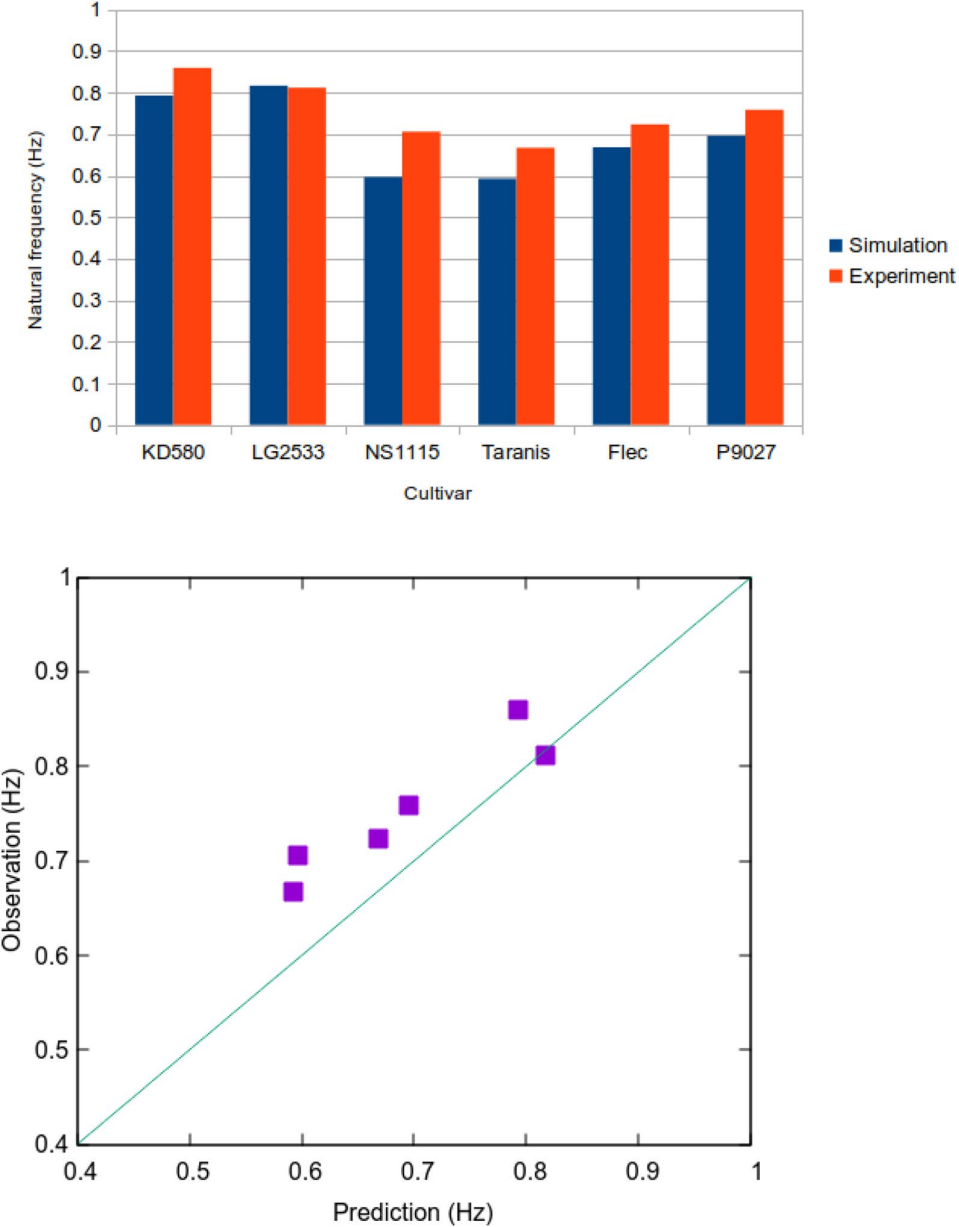
Natural frequency estimated by finite element analysis. The predicted natural frequencies of the six cultivars are mostly identical to one of the experiments.

**Figure 5:**
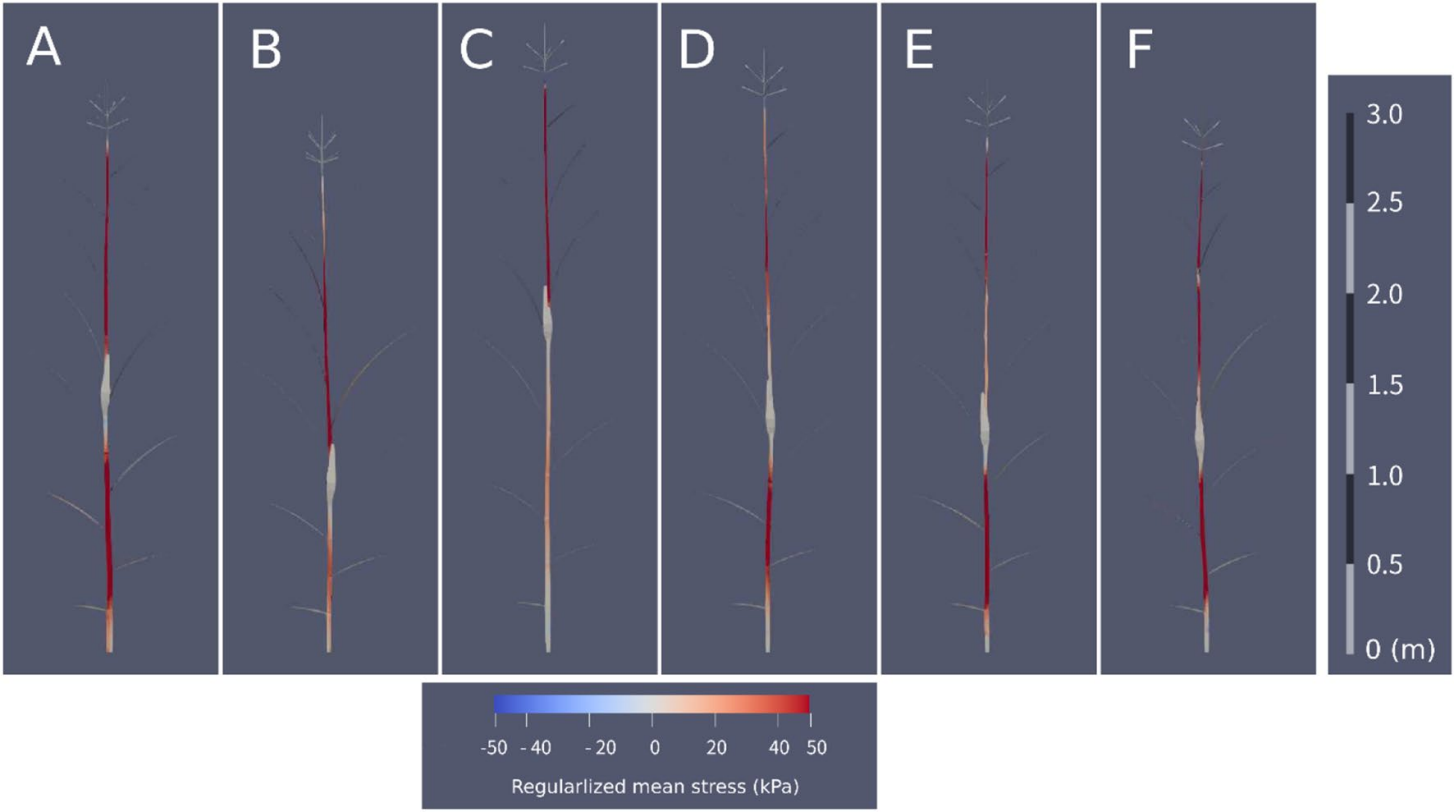
Eigenmodes simulated by finite element analysis. Fundamental modes and contour map of the regularized mean stress estimated for (**A**) KD580, (**B**) LG2533, (**C**) NS115 (**D**) Taranis, (**E**) Flec, and (**F**) P9027, respectively. The deformation vector is regularized so that the norm is equal to 1.0, and mean stress is associated with the displacement. Although the hot-spots are visible in Fig. 1A,B, the stress levels are distinctly different for each mechanical/morphological phenotype.

To verify the validity of the Young’s modulus estimated by the MEMS sensor, the natural frequencies of maize plants were predicted numerically and compared with the experimental results. Simulations were performed using a combination of the finite element and domain composition methods^38^. The results showed that the predicted Young’s modulus agreed well with the measured natural frequencies for all the cultivars (Fig. 4). Close inspection revealed that for all cultivars, except LG2533, the simulated values were approximately 3–5% lower than the measured values. Thus, although the wave velocity measured by the MEMS sensor suggests that the waves are slower than the S-waves, the error is not significant. Figure 5 shows the natural vibration modes and hot-spot locations for each cultivar. The contour map describes the mean stress corresponding to the first eigenmode for each cultivar, and the stress was higher at the positions where large deformations occurred. Figure 5A–F corresponds to the cultivars KD580, LG2533, NS115, Taranis, Flec, and P9027. Although hot-spots are visible in Fig. 1A,B, the stress levels are distinctly different for each mechanical/morphological phenotype. Two different modes, the L- and W-modes, appear in the figure, where the L-mode bends at the top, as seen in (B) and (C), while the W-mode is visible in (A) and (D–F). These observations show that if the L-mode appears in a maize plant, either the Young’s modulus at the top of the plant should be increased for that cultivar or both the top and bottom parts should be reinforced to increase lodging resistance. Thus, a combination of the MEMS sensor array and FEA can be used to develop an optimal strategy for breeding lodging-resistant cultivars.

## Discussion

The novel ultra-lightweight sensor array successfully measured the Young's modulus of corn stalks non-destructively, accurately, and at high throughput. The actual weight of each sensor head was approximately 3.5 g including fixture devices. As the averaged fresh weights of leaves, stem and apical ear of examined individuals (*n* = 48) at milk stage were 195 g, 539 g and 341 g, respectively, and the total weight of three sensors was equivalent to only 2–5% of each organs and less than 1% of total fresh weight of a single plant; therefore, the effects of installing these sensors on maize stalk was able to assume negligible. This device would also be transferable to other crops and plant species with a large above-ground structure, including *Sorghum* spp., *Miscanthus* spp., and some tree species. However, further miniaturization of the sensors would be required for application in cereal crops with shorter and thinner stem architecture such as wheat, oats and barley.

The newly developed method used an MEMS accelerometer to measure the wave velocity of surface waves in the corn stalk to estimate the Young's modulus for that section. The validation results, focusing on the natural frequencies, suggest that the wave velocity is lower than the S-wave velocity of the entity wave. Therefore, these waves are presumed to be surface waves, similar to Lamb waves.

The results of vibration tests, focusing on the step response, indicate that the theoretical damping ratio of maize is approximately 5–25%. This is significantly greater than the attenuation rates of metals and concrete; thus, entity waves may decay instantaneously. However, Lamb waves propagate through repeated reflection and refraction, and are thus transmitted farther, which would have enabled the measurement of wave velocity, as proposed in this study.

Because it is difficult to estimate the Young’s modulus of a stem from its natural frequency, the proposed MEMS sensor array is the highest-throughput measurement method for measuring the Young’s modulus of maize. The Young’s modulus was measured using MEMS sensors at two locations, the bottom and top of the ear, neither of which alone explained the natural frequency. This suggests that, similar to the shape, there is a factor that determines the natural frequency more than the Young’s modulus of the stem section, such as morphology or density. Furthermore, present study assumes that the stalks are isotropic material, however, experiments and simulations shown by the previous studies suggests that the anisotropy of the stalks characterizes the quasi-static and the dynamic behavior^39,40^. Future studies will apply the proposed methodology to the anisotropy of the stalk to identify the anisotropic material parameters.

One suggestive result is drawn from the hotspot analysis shown in Fig. 5. Figure 5 shows that the natural vibration modes differ for each cultivar, and that the stress concentration points differ even for similar morphologies. The vibration characteristics of maize can be classified into two groups based on the location of the hot spots: one group to which NS115 and LG2533 belong (single-spot) and another group to which the rest belong (double-spot). If a single-spot cultivar lodges, it should be bred to be stiffer above the ear, whereas if a double-spot cultivar falls over, it should be stiffer below the ear. Prior to this discovery, researchers found genes that made stems hard^3^; however, they did not know the locations on the stem that should be hardened. Our method shows where and how hard the stem should be.

Furthermore, this research allows for trial and error in the shape and material of corn, which is resistant to overturning. In civil and mechanical engineering, FEA has been used to design optimal shapes and materials. However, no similar approaches have been used to actively design crop morphology and physical properties to search for a grass type that is resistant to overthrow. This study proposes a high-throughput measurement technique for Young's modulus and FEA, which provides tools to search for phenotypic traits that are less susceptible to wind forces and lodging.

## Methods

### Plant materials

Six maize cultivars with varying lodging resistance: KD580, LG2533, NS115 super, Taranis, Flec, and P9027, were obtained from a local seed supplier and grown in the experimental field of Hokkaido University (43.07° N, 141.34° E) in 2022. Each plot consisting of 4 rows with 0.75 m of row spacing and 0.18 m of intra-row spacing were arranged into randomized complete block design with four replications. After land preparation by two passes of rotary tillage, followed by opening of the seed furrow at a depth of approximately 5 cm, synthetic fertilizer was applied along each row at a rate of 130 kg N ha^−1^, 180 kg P_2_O_5_ ha^−1^ and 100 kg K_2_O ha^−1^ as basal. No additional fertilizer was supplied during the growth period. Two seeds per hill were sown manually on May 10th, 2022. Atrazine-based herbicides were sprayed at 2nd leaf stage (V2) and thinned at V3 to ensure uniform stand establishment at a planting density of 74,100 plants ha^−1^. The dates of silking were recorded for each cultivar, and all measurements were performed at the milk stage (R3).

### Mechanical configuration of MEMS sensor array

Three tri-axis ultra-micro analog accelerometers (AK-KXSC9-2050, Kionix, US) modularized on 12 mm × 12 mm electric boards were connected to a PC-based data logger (Picolog 1216, Pico Technology, UK) with a 12-bit resolution and a maximum sampling frequency of 1 kHz. The detection rage and sensitivity of the accelerometers were ± 19.6 ms^−2^ and 14.9 ms^−2^/V, respectively. Only the x- and y-axis outputs were connected to the data logger with a fine copper wire having a diameter and length of 0.2 mm and 3 m, respectively. The data logger was enclosed in a plastic container and mounted on a tripod together with a laptop PC to increase field portability under the maize canopy. An application voltage of 3.3 V was applied to each accelerometer through a universal serial bus on a laptop PC coupled with a stepping-down converter module. Three accelerometers were fixed on a plastic fixture device with a heat-shrinkable tube and firmly attached to three positions along a maize stem using a plastic cable tie. Each sensor was placed on the 1st stalk node (S1), immediately below the ear node (S2), or below the uppermost leaf node (S3). All sensors were aligned horizontally to the row. The total weight of each sensor head was less than 2 g.

### Measurements of wave velocities between sensors in impact tests

**Figure 6:**
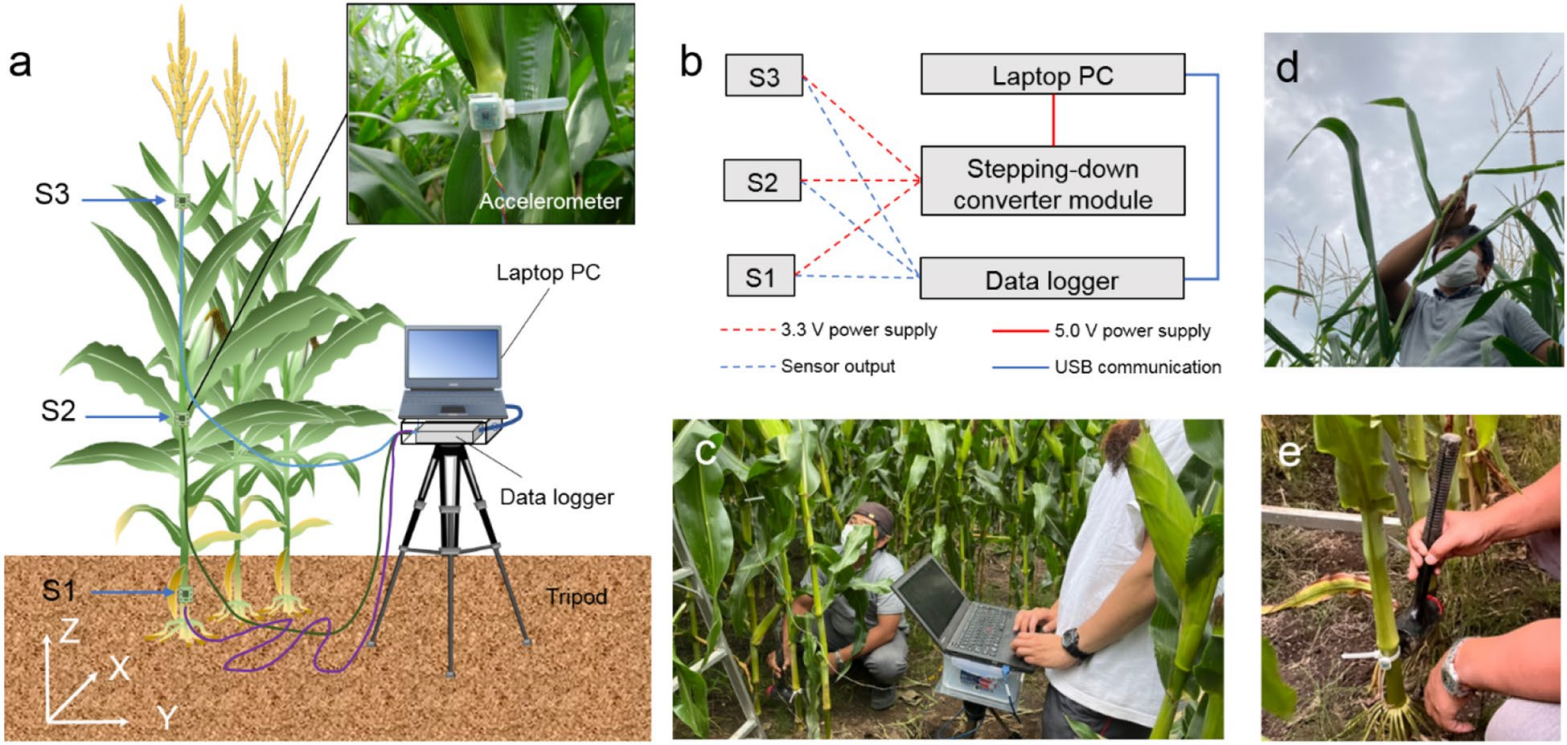
Mechanical configuration of micro-electro-mechanical systems (MEMS) sensor array. (**a**) Three accelerometers were attached to the above and lower sections of maize stalk and the output voltage was recorded using a PC-based data logger with high frequency. Traveling velocity of impact waves between S1 and S2, as well as S2 and S3 on stalk were measured. (**b**) Simplified schematic diagram of sensor array. (**c**) Field measurement of wave velocity using the developed system. (**d**) Pulling tassel base for bending vibration test. (**e**) Impact test using rubber mallet.

Young’s moduli for *E*_1_ and *E*_2_ were estimated by impact tests using the sensor array (Fig. 6). In this method, the traveling velocities of the impact wave between the S1–S2 (Vs1) and the S2–S3 intervals (Vs2) were measured, followed by destructive sampling of the corresponding stalk sections for volumetric mass density determination. The recording frequencies of the data logger were set at 1 kHz, and gentle impacts were applied several times to the 1^st^ stalk node (i.e., immediately below S1) using a rubber mallet. Measurements were conducted on two plants from the two central rows of each plot. A bending vibration test was conducted on the same plant to measure the step response. The directions of the impact and bending tests were fixed horizontally to the y-axis for all measurements. The height of each sensor was recorded at the end of the measurement.

The obtained waveform was processed and analyzed using PicoLog software Ver. 6.2.5 (Pico Technology). For the impact test, because the reproducibility of waveforms in each measurement was adequately high, a representative waveform was selected, and the arrival time for the first S-wave at each sensor was noted to calculate the traveling time between two sensors, from which Young’s modulus was derived as follows:

### Estimation of Young’s modulus based on the travel time curve

The maize plant can be considered as a linear elastic body when the deformation is small. The equation of motion can be written as:

$$\rho \frac{{\partial }^{2}u}{\partial {t}^{2}}+G\frac{{\partial }^{2}u}{\partial {x}^{2}}={f}_{s},$$

where $\rho$ is the density, $u$ is the horizontal displacement field, $t$ is the time, $G$ is the shear modulus, $x$ is the traveling distance from the bottom of the plant, and ${f}_{s}$ is the external shear force. The density of maize plants is generally non-zero; therefore, we can divide both terms by density to obtain the equation:

$$\frac{{\partial }^{2}u}{\partial {t}^{2}}+{\left({V}_{s}\right)}^{2}\frac{{\partial }^{2}u}{\partial {t}^{2}}=\frac{{f}_{s}}{\rho },$$

where

$${V}_{s}=\sqrt{\frac{G}{\rho },}$$

which is the shear velocity observed in the travel-time curve (Fig. 2). The shear modulus and Young’s modulus $E$ are related via Poisson’s ratio $\nu$ as follows:

$$G=\frac{E}{2\left(1+\nu \right)}.$$

Finally, Young’s modulus is calculated using the following equation:

$$E=2\left(1+\nu \right)\rho {\left({V}_{s}\right)}^{2}.$$

### FEA

We used modal analysis based on the domain composition method^38,41–43^. The geometry of maize was approximated using functional structural plant models^44–47^ and each node was converted into finite element meshes. The finite element meshes were partially overlapped and used for modal analysis. The governing equations of the system are as follows:

$$\rho \frac{{\partial }^{2}{u}_{i}}{\partial {t}^{2}}+\frac{\partial {\sigma }_{ij}}{\partial {x}_{j}}={b}_{i},$$

where ${\sigma }_{ij}$ is a Cauchy stress tensor, ${x}_{j}$ is the coordinate, and ${b}_{i}$ is a body force. The equation is discretized based on the FEM, which results in:

$${M}_{IJ}\frac{{\partial }^{2}{u}_{J}}{\partial {t}^{2}}+{K}_{IJ}{u}_{J}={F}_{I},$$

where ${M}_{IJ}$ is the mass matrix, ${u}_{J}$ is the discretized displacement field, ${K}_{IJ}$ is the stiffness matrix, and ${F}_{I}$ is the external force vector. Under free vibration, the solution is expressed as

$${u}_{J}={U}_{J}\left(\omega \right)exp\left(-i\omega t\right)$$

Substituting this equation into the discretized equation, the following generalized eigen value problem is obtained

$${\omega }^{2}{M}_{IJ}{U}_{J}-{K}_{IJ}{U}_{J}=0$$

The maize morphology was captured by a 3D scanner and generated by fitting a FEM mesh to a 3D point cloud. For maize stalks and maize leaves, all individuals were measured for length, thickness, thickness, and angle for each cultivar, and the average values were used to generate the FE mesh. 8-node 3D solid elements are used for the FE-model. The boundary condition is a fixed boundary at the ground edge and free boundaries in other areas. The Poisson's ratio 0.3 was employed for the plant body, considering that the Poisson’s ratio of dry plant organs is around 0.15–0.2^48,49^, and flesh ones are around 0.27–0.38 (2008). Furthermore, it is worth noting that FEA uses Young's modulus and density in the S1–S2 and S2–S3 sections according to the experimental values.

### Experimental estimation of natural frequency

A maize plant was approximated as a single degree of freedom system as proposed by Baker et al.^50^,

$$m\frac{{\partial }^{2}u}{\partial {t}^{2}}+c\frac{\partial u}{\partial t}+ku=f$$

where $m$ is the concentrated mass, $u$ is the horizontal displacement, $c$ is the damping coefficient, $k$ is the spring coefficient, and $f$ is the external force. The analytical solution of the equation under free vibration is given by:

$$u\left(t\right)=Aexp\left(-h\omega t\right)cos\left(\omega \sqrt{1-{h}^{2}}t-\psi \right),$$

where $A$ is an amplitude, and

$$\omega =\sqrt{\frac{k}{m}} .$$

We conducted an impact test and measured the step response of the maize plant in the horizontal direction. Parameters $h$ and $\omega$ were identified by fitting the analytical solution to the output wave using the stochastic gradient descent method^51^.

### Statistical analysis

Data for the impact and bending vibration tests were analyzed using the open-access statistical tool for agricultural research (STAR ver. 2.0.1; International Rice Research Institute, 2014) in R package (ver.3.3.1). One-way analysis of variance was performed for comparisons among cultivars, followed by Tukey’s honestly significant difference test for pairwise mean comparisons at *p* < 0.05. Student’s t-test was performed to compare the Young’s modulus, wave velocity, and volumetric mass density of the below- and above-ear stalk sections.

### Legality statement

The plant materials examined in the present study are commercially available hybrids purchased or provided from local seed suppliers with permissions for experimental use. This study complied with institutional, national, and international legislation and guidelines for the use of all the materials and experimental methods.
